# Effects of circadian clock and light on melatonin concentration in *Hypericum perforatum* L. (St. John’s Wort)

**DOI:** 10.1186/s40529-020-00301-6

**Published:** 2020-09-15

**Authors:** Ming-Hsiu Chung, Tzu-Shing Deng

**Affiliations:** 1 Taiwan Sugar Corporation, Nanjing Farm, Shuishang Township, Chiayi County 608 Taiwan R.O.C.; 2 Department of Agronomy, National Chung-Hsing University, 145 Xingda Rd, South District, Taichung City, 40227 Taiwan R.O.C.

**Keywords:** Circadian clock, *Hypericum perforatum* L, Medicinal herb, Melatonin, Entrained, Light

## Abstract

**Background:**

Melatonin acts as a signaling hormone and entraining agent in many organisms. We studied the spatiotemporal regulation and influence of light (photoperiods, intensities, and spectral qualities) on melatonin concentration in the medicinal herb *Hypericum perforatum* L. Furthermore, melatonin concentrations in the leaves of eight species of the *Hypericum* genus were compared and analyzed using high-performance liquid chromatography.

**Results:**

Melatonin concentration was found to be the highest in its flowers and leaves. The leaves exhibited a rhythmic variation in melatonin concentration of approximately 24 h under both light–dark entrained (Zeitgeber time) and constant light [circadian time (CT)] conditions, with melatonin concentration peaking at approximately CT6 in the middle of the subjective day. Melatonin concentration was influenced significantly by not only photoperiods but also applied light’s wavelength and intensity. It was approximately six times higher under long-day conditions (18-h light:6-h dark) than under short-day photoperiods (10-h light:14-h dark) and was the highest (131 μg/g fresh weight [FW]) under treatment with blue light at an intensity of 45 µmol·m^2^/s of photons. The melatonin concentration of the two examined *Hypericum spp.*, namely *H. kouytchense* Lev. and *H. coris* L., were approximately twice that of *H. perforatum* L.

**Conclusion:**

Our findings provide first insights on melatonin-related functions and mechanisms in the circadian system of *H. perforatum* and useful resources for further melatonin-oriented research and possible applications in agriculture and pharmaceutical industries.

## Background

Melatonin, a highly conserved molecule during evolution, is ubiquitously present in living organisms, ranging from bacteria to mammals (Hardeland et al. 1995; Hardeland and Poeggeler 2003; Hardeland et al. 2011; Hardeland 2019; Zhao et al. 2019). It was first isolated from amphibian skin and characterized to have a role in amphibian skin coloration (Lerner et al. 1958). In animals, melatonin is an indoleamine hormone, synthesized by the pineal gland from tryptophan, its basic precursor (Murch et al. 2000; Murch and Saxena 2006).

Melatonin has been found to be involved in numerous different processes in all organisms investigated so far. It exhibits pleiotropic biological activities in phylogenetically diverse species. Some of these activities are mediated by G-protein-coupled and nuclear receptors (Reppert 1997; Dubocovich et al. 1999; Imbesi et al. 2009), whereas others are receptor-independent, such as melatonin’s interactions with free radicals and activities mediated by its bioactive metabolites (Reiter 1996; Tan et al. 2002; Schaefer and Hardeland 2009). A common characteristic in all the pleiotropic functions of melatonin is that it is involved in setting the timing of all processes (Cassone and Natesan 1997).

The endogenous circadian clock, which controls daily rhythmic output processes, is maintained in constant laboratory conditions with a period of approximately 24 h in almost all phyla—from microorganisms to humans (Aschoff 1965; Pittendrigh 1993). These 24-h oscillations (Roenneberg and Morse 1993) under a light–dark cycle drive the rhythm or timing of varied biological activities—from bioluminescence, enzyme activities, and phototactic movements in unicellular algae (Balzer and Hardeland 1991; Roenneberg et al. 1989; Deng and Roenneberg 1997; Deng and Roenneberg 2002; Aiyar et al. 2017; Deng 2018) to the sleep–wake cycle in humans (Wever 1979). Melatonin plays a key role in circadian rhythms and in seasonal photoperiodic regulation in animal systems (Hardeland et al. 1993; Foulkes et al. 1997). Other than that in vertebrates, melatonin was first discovered in unicellular photosynthetic marine algae (Poeggeler et al. 1991) and then in plant species, such as some edible plants (Dubbels et al. 1995) and medicinal herbs (Murch et al. 1997; Chen et al. 2003).

*Hypericum perforatum* L. (St. John’s wort), a prominent perennial medicinal herb, has been widely used in the treatment of mild-to-moderate depression (Gaster and Holroyd 2000) and as a potential source for anticancer and antiviral medicines (Schempp et al. 2002; Pasqua et al. 2003). Melatonin is present in *Hypericum perforatum* L. (Murch et al. 1997; Murch and Saxena 2006), but its daily localization, possible role, and regulation mechanism remain largely unknown.

In this study, we investigated the influence of the circadian clock and the main zeitgeber, light, on melatonin concentration and its spatial distribution in *H. perforatum* L. as well as the interspecific differences in melatonin concentrations among various species of *Hypericum*.

## Materials and methods

### Plant materials

All seeds of *H. perforatum* L. and other *Hypericum* species (*H. olympicum* L., *H. hookerianum*, *H. montanum* L., *H. fragile*, *H. coris* L., *H. kouytchense* Lev., and *H. formosanum*) were provided by Thomas Bopp (Technical Director of the Botanical Garden of Friedrich-Schiller University, Jena, Germany) or purchased from Flecke Saaten Handel GMBH (Wunstorf, Germany). The seeds were washed three times with distilled water and stratified in refrigerator storage for at least 2 weeks before germination (Briskin and Gawienowski 2001). Plants were grown in pots containing a 25:1:1 (w/w/w) mixture of soil, vermiculite, and perlite in either a growth chamber (Lian Shen Enterprise, Taichung, Taiwan)—with growth conditions of 18-h light:6-h dark (except experiments with different photoperiods) and fluorescent light = 125 µmol·m^2^/s photons—or a greenhouse—with growth conditions of 18-h light:6-h dark with eight different LED lights [CW (cool white light, 5000 K), WW (warm white light, 2700 K), 9B (450 nm), 9R (660 nm), 7R1G1B (R:G:B:IR ratio = 82:9:9:0), 8R1B (R:G:B:IR ratio = 91:0:9:0), 3R3B3FR (R:G:B:IR ratio = 49:1:33:17), and 9FR (730 nm); 9–90 µmol·m^2^/s photons; Model: NBL-MEL-24; Nano Bio Light, Taiwan] at 22 °C, with plants watered three times a week with distilled water.

### Melatonin extraction

Plant materials were harvested at designated stages for the experiments (for Fig. 2, the cultivating days approximately 180 days till plants in bloom; the average fresh weights of the root, stem, leaf, and flower parts after harvesting were approximately 4, 8, 8, and 0.5 g, respectively; the plant age was approximately 60 days before harvest for the others); they were separated into root, stem, leaf, and flower parts and maintained at −60 °C for further measurement of melatonin concentration. Melatonin standard and butylated hydroxytoluene were purchased from Sigma (St. Louis, MI, USA). Other organic solvents were obtained from Merck. Plant parts for various treatments were weighed, frozen in liquid nitrogen, and ground to a fine powder using a glass rod. The powdered material (0.3 g) was first extracted in 10 mL of 50 mM sodium phosphate buffer (pH 8) containing 5 µM butylated hydroxytoluene as an antioxidant through ultrasonication (Branson 1200) for 45 min in darkness with minimal shaking. The insoluble material was further washed twice with 1 mL of 50 mM sodium phosphate buffer (pH 8). The eluant was combined with the aforementioned extracts. The supernatant was obtained through centrifugation (Universal 32 R; Hettich, Germany) at 4 °C at 5000 rpm for 10 min and then filtered (90 mm, No. 1; Advantec, Japan) in vacuum to prepare for further purification. Extracts were purified twice using 12 mL of ethyl acetate through phase partition. The combined organic phases were evaporated to dryness in vacuum. The dry residue was redissolved in 1 mL of methanol retained in a 2-mL brown Eppendorf tube. The supernatant was obtained through centrifugation (5415D; Eppendorf, Hamburg) at 13,000 rpm for 5 min and then passed through a Millipore membrane (0.2 μm, PL-6054540; PALL) for further analysis (Guerrero et al. 2001).

### Qualitative and quantitative analysis of melatonin

**Fig. 1:**
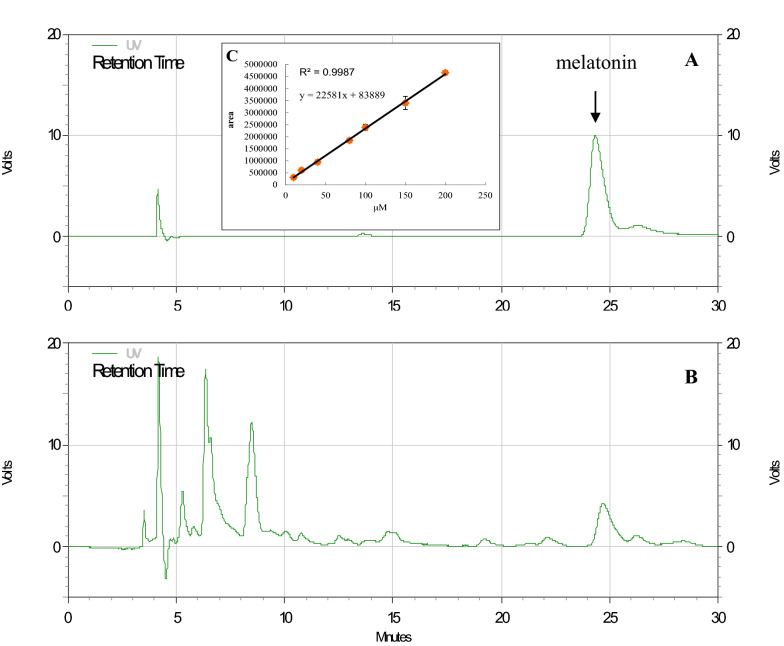
HPLC analysis for melatonin: **a** melatonin standard (100 μM), **b** *H. perforatum* L. extract, and **c** calibration curve of melatonin standard. Data are the mean of three replicates

The melatonin standard was weighed and dissolved in 1 mL of methanol to obtain serial concentrations. Three injections were performed for each dilution. The standard curve was calibrated using the linear least-squares regression equation derived from the peak area (r^2^ > 0.99; inset, Fig. 1a). Melatonin was quantified through HPLC (model L2600, Hitachi; Mightysil RP-18 GP 250-4.6 (5 μm), Kanto Chemical). The mobile phase was water/acetonitrile/acetic acid (82:16.5:1.5, v/v/v), with a flow rate of 0.7 mL/min at 40 °C and detection at 280 nm (Hernandez-Ruiz et al. 2004).

### Statistical analysis

Data were compared using one-way analysis of variance followed by Fisher’s least significant difference test (*P *< 0.05, n = 3).

## Results

### Qualitative and quantitative measurements and spatial distribution of melatonin in *H. perforatum* L.

**Fig. 2:**
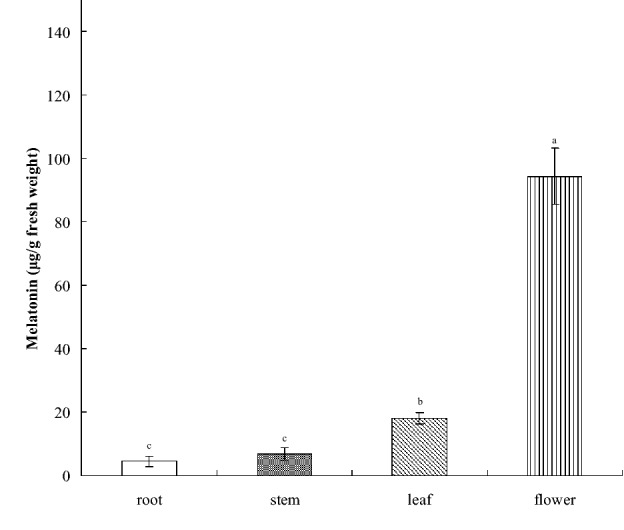
Melatonin concentrations in various parts of *H. perforatum* L. Data are the mean of three replicates, and different letters represent significant differences at *p *< 0.05

Established reliable measurements (see inset, r^2^ = 0.9987) for melatonin concentration in *Hypericum perforatum* L. were qualitatively and quantitatively determined through high-performance liquid chromatography (HPLC; Fig. 1). Melatonin concentration in different tissues and organs of *H. perforatum* L. was then analyzed (Fig. 2). Melatonin concentration was the highest in the flowers (approximately 100 μg/g FW), followed by that in the leaves (approximately 20 μg/g FW), with the stem and roots having the lowest concentration (< 10 μg/g FW; *p* < 0.05).

### Temporal regulation and mechanism of melatonin concentration in *H. perforatum* L.

**Fig. 3:**
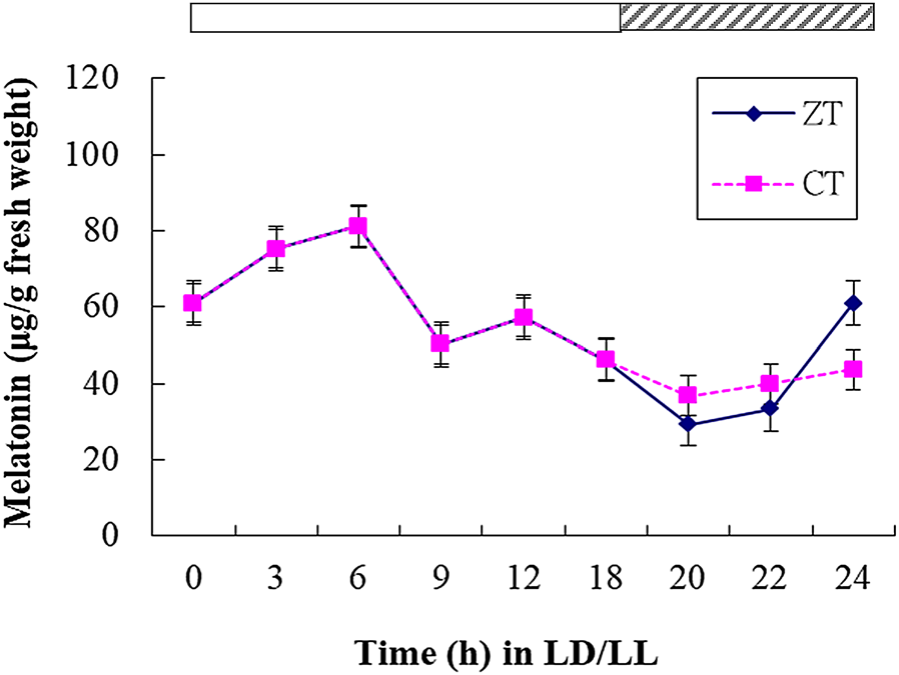
Rhythmic changes in melatonin concentrations in *H. perforatum* L. leaves with time under 18-h light:6-h dark photoperiodic cycles (*Zeitgeber* time, ZT; solid diamonds) and constant light conditions (circadian time, CT; solid squares). The white bar at the top indicates the light period. The black-shaded bar at the top indicates the dark period and the subjective night. Data are the mean of three replicates

The oscillation of melatonin concentration in the leaves of *H. perforatum* L. entrained to an 18-h light: 6-h dark [Zeitgeber time (ZT)] and transferred to constant light [circadian time (CT)] conditions was measured. We observed that a melatonin rhythm with rather modest amplitude was controlled by the circadian clock, peaking at approximately CT6 in the middle of the subjective day (Fig. 3).

### Effects of photoperiods on melatonin concentration in *H. perforatum* L.

**Fig. 4:**
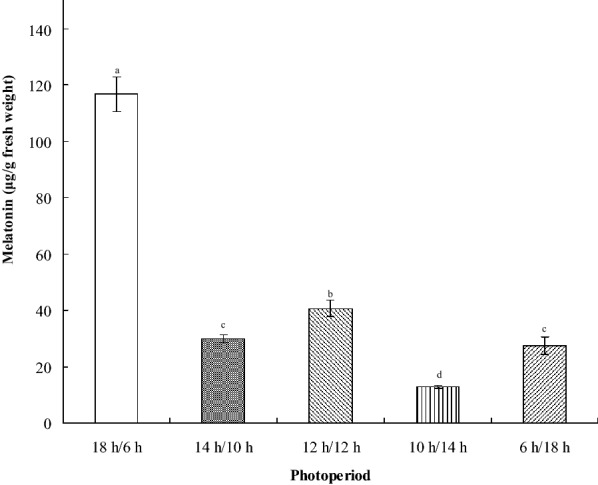
Melatonin concentrations of *H. perforatum* L. leaves harvested at ZT6 for extraction for various photoperiods. Data are the mean of three replicates, and different letters represent significant differences at *p *< 0.05

The influence of different photoperiods on the melatonin concentration in *H. perforatum* L. leaves was also investigated. The highest melatonin concentration (approximately 120 μg/g FW) was found at ZT6 under long-day conditions (18-h light:6-h darkness), whereas the lowest melatonin concentration (< 20 μg/g FW) was found under short-day conditions (10-h light:14-h dark; Fig. 4). Thus, melatonin concentration is the highest under summer day conditions.

### Effects of light quality and intensity on melatonin concentration in *H. perforatum* L.

**Fig. 5:**
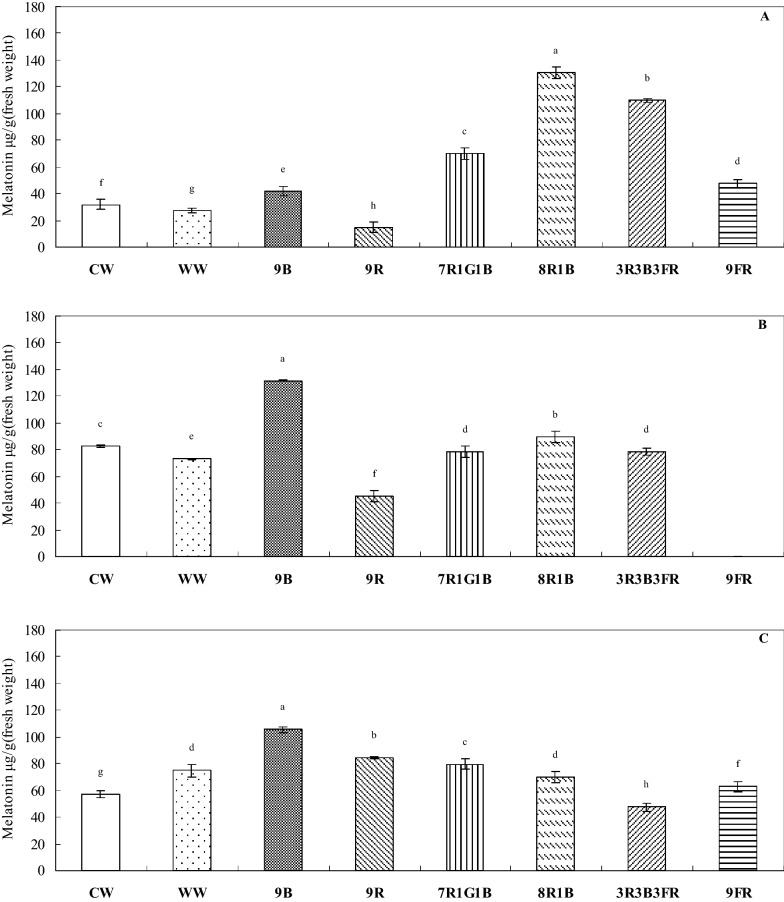
Effects of light quality and intensity on melatonin concentration in *H. perforatum* L. **a** level F (approximately 90 µmol·m^2^/s photons), **b** level 5 (approximately 45 µmol·m^2^/s photons), and **c** level 1 (approximately 9 µmol·m^2^/s photons). The eight LED light sources are indicated as CW (cool white light, 5000 K), WW (warm white light, 2700 K), 9B (450 nm), 9R (660 nm), 7R1G1B (R:G:B:IR ratio = 82:9:9:0), 8R1B (R:G:B:IR ratio = 91:0:9:0), 3R3B3FR (R:G:B:IR ratio = 49:1:33:17), and 9FR (730 nm). Data are the mean of three replicates, and different letters represent significant differences at *p *< 0.05

Eight wavelengths (six monochromatic and three mixed-light sources) and three intensities (9, 45, and 90 µmol·m^2^/s photons) were used to explore the effects of light quality and intensity on melatonin concentration in *H. perforatum* L. leaves under 18-h light:6-h dark conditions using LED lights (NBL-MEL-24; Nano Bio Light, Taiwan). Of these, treatment with 9B (monochromatic blue light, 450 nm) at level 5 (45 µmol·m^2^/s photons) induced the highest melatonin concentration (approximately 131 μg/g FW; Fig. 5b), whereas the lowest concentration (< 20 μg/g FW) was found for treatment with 9R (monochromatic red light, 660 nm) at level F (90 µmol photons·m^2^/s; Fig. 5a). The 8R1B treatment (R:G:B:IR ratio = 91:0:9:0) at level F induced the second highest concentration (approximately 126 μg/g FW) of melatonin in *H. perforatum* L. leaves (Fig. 5a).

### Biodiversity and interspecific difference of melatonin concentration among *Hypericum* spp.

**Fig. 6:**
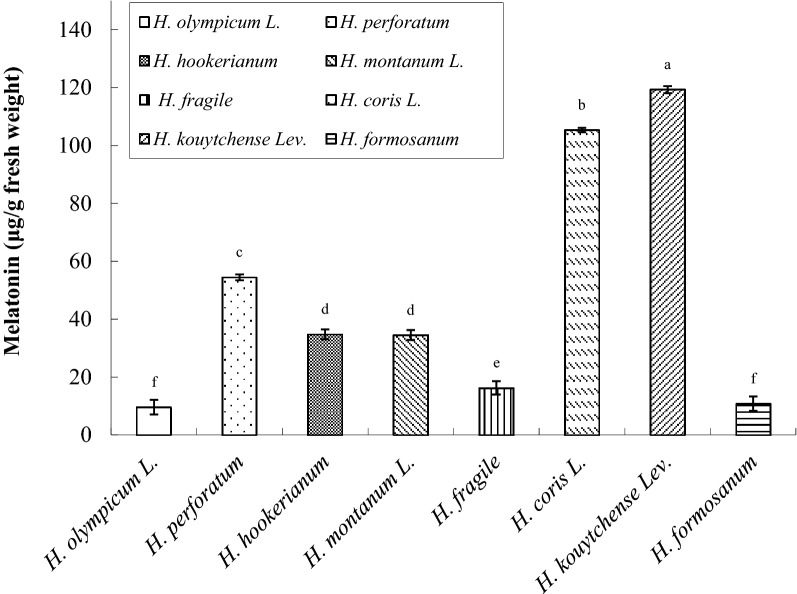
Comparison of melatonin concentration in leaves of eight *Hypericum* spp. Data are the mean of three replicates, and different letters represent significant differences at *p* < 0.05

Understanding the differences in melatonin concentration among various *Hypericum* spp. may provide knowledge on their potential medicinal uses. Therefore, we compared melatonin concentration in the leaves of eight *Hypericum* spp.. The selected species exhibited highly variable melatonin concentrations at ZT6 under the 18-h light:6-h dark cycle (Fig. 6). Of them, *H. kouytchense* Lev. and *H. coris* L. exhibited a leaf melatonin concentration of approximately two times (119 and 105 μg/g FW, respectively) that of *H. perforatum* L. (54 μg/g FW; Fig. 6).

## Discussion

Circadian clock mechanisms have been investigated in single neurons (Michel et al. 1993), unicellular algae (Roenneberg and Deng 1997), microorganisms (Merrow et al. 1999), and higher plants (Greenham and McClung 2015; Greenham et al. 2017). All components of this endogenous clock occur at the cellular level. This complex temporal program is a prerequisite for specialization, fitness, and survival within the chrono-ecological niches of the “temporal–space” day and enables organisms to thrive by integrating environmental changes with endogenous circadian systems (Roenneberg et al. 1995).

As mentioned, melatonin is a highly conserved molecule during evolution and exerts many known pleiotropic biological activities among diverse species. Studies have revealed new functions and involvement in physiological and biochemical mechanisms of melatonin (e.g., proinflammatory and anti-inflammatory networks by noncoding RNAs) (Hardeland 2019).

Melatonin concentration in *H. perforatum* is higher than that in most of the other plants (Murch et al. 1997; Murch and Saxena 2006; Deng 2018; Erland et al. 2018). In this study, reliable measurements of melatonin concentration in *H. perforatum* were determined qualitatively and quantitatively through HPLC (Additional file 1: Fig. S1 and Additional file 2: Fig. S2). Melatonin concentration in *H. perforatum* was the highest in the flowers, followed by that in the leaves—thus indicating a tissue–organ differential expression. This also reflects both spatial distribution and regulation of the signaling hormone and conventional usage of *H. perforatum* as “materia medica” for treating minor or moderate forms of depression and a range of mood disorders (Heinrich and Anagnostou 2017). Melatonin acts as a chronobiotic agent, and treatment with melatonin can entrain circadian rhythms in most visually impaired people and night-shift workers who have free-running rhythms (Sack and Lewy 1997; Sack et al. 2000); it exerts clinically beneficial effects on circadian rhythm–related sleep disorders, jetlag, and even Alzheimer disease (Zisapel 2018). Melatonin treatment was recently found to attenuate sepsis mediated myocardial depression by modulating BAP31 pathways and improve cardiac performance by inhibiting mitochondrial injury and endoplasmic reticulum dysfunction (Zhang et al. 2020).

Melatonin levels are subject to circadian control in human (Czeisler et al. 1999; Lockley et al. 2003) and other animal (Tosini and Menaker 1996; Heigl and Gwinner 1995) systems as well as in the photosynthetic marine dinoflagellate *Lingulodinium* (Hardeland 1993; Hardeland et al. 1995) and numerous chlorophycean microalgae (Kolář and Macháčková 2005). However, only a few plants, such as *Chenopodium rubrum* (Lecharny and Wagner 1984) and rice (*Oryza sativa*) seedlings (Byeon and Back 2016), demonstrate this phenomenon. Our results demonstrated that the circadian melatonin rhythm in *H. perforatum* peaks at approximately CT6 in the middle of the subjective day. Because of their sessile nature, plants encounter more environmental challenges than animals. Phytomelatonin is a multi-regulatory molecule with diverse functions in growth and development; protection against stresses, such as reactive oxygen species and other free radicals, high ambient temperature, and ultraviolet (UV) radiation; and photosynthesis inhibition (Arnao and Hernandez-Ruiz 2015; Zhao et al. 2019). Our findings might coordinate the circadian maximal amplitude of melatonin in *H. perforatum* and yield potentially protective effects against daily rhythmic environmental stresses.

Garner and Allard (1920) examined the effects of maximum daily light exposure on growth and sexual reproduction (flowering) in crops (e.g., soybeans and tobacco) and introduced the term “photoperiodism” to indicate the response of organisms to the relative length of day and night (i.e., photoperiod) (Garner and Allard 1920). The photoperiodic mechanism was initially proposed for plants but was later identified in insects on the basis of the circadian system (Bünning 1936). More modern models have combined external and internal coincidence hypotheses to suggest that photoperiodic induction is a function of the circadian system (Pittendrigh 1981; Pittendrigh 1993; Sauders 2016). Our current results clearly demonstrate that the melatonin concentration in *H. perforatum* followed a photoperiodic response: Under long-day conditions (18-h light:6-h dark), it reached a maximum concentration of approximately 120 μg/g FW—nearly six times higher than that in a short-day photoperiod (10-h light:14-h dark). The photoperiodic induction of melatonin might also reflect the original natural habitat of *H. perforatum* (a long-day plant) and the regulatory function of the circadian system.

As a signaling and entraining agent, melatonin is sensitive to environmental changes, particularly to the strong Zeitgeber, light. The human circadian melatonin rhythm is sensitive and can be reset by short-wavelength monochromatic light (460 nm), which demonstrates twice as much melatonin suppression as does 555-nm monochromatic light. The effects depend on exposure duration in addition to spectral quality (Lockley et al. 2003). Photoperiodic green light (522 nm) can accelerate chick embryo development and alter hatch-related hormones, resulting in earlier hatching, probably because of changes in melatonin rhythm (Tong et al. 2018). Melatonin was found in the roots of *Glycyrrhiza uralensis*, which varied in concentration in response to the spectral quality of light including red and blue light as well as white light (control) and UV-B radiation, with highest melatonin concentration noted in the plants exposed to red light (Afreen et al. 2006). In the present study, treatment with 9B (450 nm) at level 5 (45 µmol·m^2^/s photons) maximized the melatonin concentration in *H. perforatum* leaves, whereas treatment with 9R (660 nm) at level F (90 µmol·m^2^/s photons) minimized it.

The temperate areas worldwide feature approximately 450 species of *Hypericum* (Robson 2003). Nevertheless, *H. perforatum* L. remains the most widely used medicinal herb in the *Hypericum* genus for academia and industry thus far, owing to its evidence-based therapeutic effects against depression and other potential uses (Schempp et al. 2002; Pasqua et al. 2003; Ernst 2003; Deng 2018). Melatonin is more abundant in *H. perforatum* than in most of the other plants (Murch et al. 1997; Murch and Saxena 2006; Erland et al. 2018) and is involved in many integrative physiological processes, including possible protection against stress (Tan et al. 2011), photoperiodic response, and the circadian system. Notably, the melatonin concentrations of two species in our *Hypericum* collection, namely *H. kouytchense* Lev. and *H. coris* L., were twice that in *H. perforatum* L.

## Conclusion

Melatonin features unraveled functions and has exhibited novel physiological applications. In this study, melatonin found to be the most abundant in flowers and leaves of *H. perforatum* L., indicating the regulation of the spatial distribution with a tissue–organ differential expression. In contrast to the circadian rhythmicity of melatonin in animals, the melatonin concentration in *H. perforatum* peaked in the middle of the subjective day for alleviating possible environmental stresses. Melatonin levels in *H. perforatum* exhibited notable photoperiodic responses, which could be regulated by light wavelength and intensity. Although hypericin is an effective natural photoactive pigment or fluorophore in *Hypericum perforatum* L. (Kazemi et al. 2012), the data on photoreceptors related to melatonin regulation in this herb are limited. Here, we speculate that this process might involve certain blue photoreceptors, such as cryptochrome and phototropin. We also compared interspecific differences in melatonin concentration in eight *Hypericum* spp.. The melatonin-rich species of *Hypericum* is a highly valuable finding from our study. Taken together, we elucidated the potential functions of melatonin in the circadian system of *H. perforatum* and provided future directions for melatonin-oriented research and applications in agriculture and pharmaceutical industries and their related fields.

## Supplementary information

**Additional file 1: Figure S1.** HPLC analysis of melatonin standard.

**Additional file 2: Figure S2.**  UV spectrum for melatonin standard in HPLC.

## References

[CR1] Afreen F, Zobayed SMA, Kozai T (2006). Melatonin in *Glycyrrhiza uralensis*: response of plant roots to spectral quality of light and UV-B radiation. J Pineal Res.

[CR2] Aiyar P, Schaeme D, Garcia-Altares M, Flores DC, Dathe H, Hertweck C, Sasso S, Mittag M (2017). Antagonistic bacteria disrupt calcium homeostasis and immobilize algal cells. Nature Commun.

[CR3] Arnao MB, Hernandez-Ruiz J (2015). Functions of melatonin in plants: a review. J Pineal Res.

[CR4] Aschoff J (1965). Circadian rhythms in man. Science.

[CR5] Balzer I, Hardeland R (1991). Circadian rhythmicity in the stimulation of bioluminescence by biogenic amines and MAO inhibitors in *Gonyaulax polyedra*. Int J Biometeorol.

[CR6] Briskin DP, Gawienowski MC (2001). Differential effects of light and nitrogen on production of hypericins and leaf glands in *Hypericum perforatum*. Plant Physiol Biochem.

[CR7] Bünning E (1936). Die endogene Tagesrhythmik als Grundlage der photoperiodischen Reaktion. Ber dtsch Bot Ges.

[CR8] Byeon Y, Back K (2016). Low melatonin production by suppression of either serotonin N-acetyltransferase or N-acetylserotonin methyltransferase in rice causes seedling growth retardation with yield penalty, abiotic stress susceptibility, and enhanced coleoptile growth under anoxic conditions. J Pineal Res.

[CR9] Cassone VM, Natesan AJ (1997). Time and time again: the phylogeny of melatonin as a transducer of biological time. J Biol Rhythms.

[CR10] Chen G, Huo Y, Tan DX, Liang Z, Zhang W, Zhang Y (2003). Melatonin in Chinese medicinal herbs. Life Sci.

[CR11] Czeisler CA, Duffy JF, Shanahan TL, Brown EN, Mitchell JF, Rimmer DW, Ronda JM, Silva EJ, Allan JS, Emens JS, Dijk D-J, Kronauer RE (1999). Stability, precision, and near-24-hour period of the human circadian pacemaker. Science.

[CR12] Deng TS (2018). Biological clocks, some clock-related diseases, and medicinal plants. PsyCh J.

[CR13] Deng TS, Roenneberg T (1997). Photobiology of the *Gonyaulax* circadian system. II. Allopurinol inhibits blue light effects. Planta.

[CR14] Deng TS, Roenneberg T (2002). The flavo-enzyme xanthine oxidase is under circadian control in the marine alga *Gonyaulax*. Naturwissenschaften.

[CR15] Dubbels R, Reiter RJ, Klenke E, Goebel A, Schnakenberg E, Ehlers C, Schiwara HW, Schloot W (1995). Melatonin in edible plants identified by radioimmunoassay and high-performance liquid chromatograph-mass spectrometry. J Pineal Res.

[CR16] Dubocovich ML, Masana MI, Benloucif S (1999) Molecular pharmacology and function of melatonin receptor subtypes. In: Olcese J (ed) Melatonin after four decades: an assessment of its potential. Adv Exp Med Biol 460:181–190 10810513

[CR17] Erland LAE, Saxena PK, Murch SJ (2018). Melatonin in plant signalling and behaviour. Funct Plant Biol.

[CR18] Ernst E (2003). *Hypericum*: The genus *Hypericum*.

[CR19] Foulkes NS, Borjigin J, Snyder SH, Sassone-Corsi P (1997). Rhythmic transcription: the molecular basis of circadian melatonin synthesis. Trends Neurosci.

[CR20] Garner WW, Allard HA (1920). Effect of the relative length of day and night and other factors of the environment on growth and reproduction in plants. J Agric Res.

[CR21] Gaster B, Holroyd J (2000). St. John’s wort for depression–A systematic review. Arch Intern Med.

[CR22] Greenham K, McClung CR (2015). Integrating circadian dynamics with physiological processes in plants. Nature Rev Genet.

[CR23] Greenham K, Guadagno CR, Gehan MA, Mockler TC, Weinig C, Ewers BE, McClung CR (2017). Temporal network analysis identifies early physiological and transcriptomic indicators of mild drought in *Brassica rapa*. Elife.

[CR24] Guerrero JR, García-Ruíz P, Sánchez-Bravo J, Acosta M, Arnao MB (2001). Quantitation of indole-3-acetic acid by LC with electrochemical detection in etiolated hypocotyls of *Lupinus albus*. J Liq Chromatogr Rel Tech.

[CR25] Hardeland R (1993). The presence and function of melatonin and structurally related indoleamines in a dinoflagellate, and a hypothesis on the evolutionary significance of these tryptophan-metabolites in unicellulars. Experientia.

[CR26] Hardeland R (2019). Aging, melatonin, and the pro- and anti-inflammatory networks. Int J Mol Sci.

[CR27] Hardeland R, Poeggeler B (2003). Non-vertebrate melatonin. J Pineal Res.

[CR28] Hardeland R, Reiter RJ, Poeggeler B, Tan DX (1993). The significance of the metabolism of the neurohormone melatonin- antioxidative protection and formation of bioactive substances. Neurosci Biobehav Rev.

[CR29] Hardeland R, Balzer I, Poeggeler B, Fuhrberg B, Uria H, Behrmann G, Wolf R, Meyer TJ, Reiter RJ (1995). On the primary fuctions of melatonin in evolution: mediation of photoperiodic signals in a unicell, photooxidation, and scavenging of free radicals. J Pineal Res.

[CR30] Hardeland R, Cardinali DP, Srinvivasan V, Spence DW, Brown GM, Pandi-Perumal SR (2011). Melatonin-a pleiotropic, orchestrating regulator molecule. Prog Neurobiol.

[CR31] Heigl S, Gwinner E (1995). Synchronization of circadian rhythms of house sparrows by oral melatonin: effects of changing period. J Biol Rhythms.

[CR32] Heinrich M, Anagnostou S (2017). From Pharmacognosia to DNA-based medicinal plant authentication – pharmacognosy through the centuries. Planta Med.

[CR33] Hernandez-Ruiz J, Cano A, Arnao MB (2004). Melatonin: a growth stimulating compound present in lupine tissues. Planta.

[CR34] Imbesi M, Arslan AD, Yildiz S, Sharma R, Gavin D, Tun N, Manev H, Uz T (2009). The melatonin receptor MT1 is required for the differential regulatory actions of melatonin on neuronal ‘clock’ gene expression in striatal neurons in vitro. J Pineal Res.

[CR35] Kazemi SY, Abedirad SM, Zali SH, Amiri M (2012). Hypericin from St. John’s Wort (*Hypericum perforatum*) as a novel natural fluorophore for chemiluminescence reaction of bis (2,4,6-trichlorophenyl) oxalate-H_2_O_2_-imidazole and quenching effect of some natural lipophilic hydrogen peroxide scavengers. J Lumines.

[CR36] Kolář J, Macháčková I (2005). Melatonin in higher plants: occurrence and possible functions. J Pineal Res.

[CR37] Lecharny A, Wagner E (1984). Stem extension rate in light-grown plants - Evidence for an endogenous circadian rhythm in *Chenopodium rubrum*. Physiol Plant.

[CR38] Lerner AB, Case JD, Takahashi Y, Lee TH, Mori W (1958). Isolation of melatonin, the pineal gland factor that lightens melanocytes. J Am Chem Soc.

[CR39] Lockley SW, Brainard GC, Czeisler CA (2003). High sensitivity of the human circadian melatonin rhythm to resetting by short wavelength light. J Clin Endocrinol Metab.

[CR40] Merrow M, Brunner M, Roenneberg T (1999). Assignment of circadian function for the *Neurospora* clock gene frequency. Nature.

[CR41] Michel S, Geusz ME, Zaritsky JJ, Block GD (1993). Circadian rhythm in membrane conductance expressed in isolated neurons. Science.

[CR42] Murch SJ, Saxena PK (2006). A melatonin-rich germlasm line of St. John’s wort (*Hypericum perforatum* L.). J Pineal Res.

[CR43] Murch SJ, Simmons CB, Saxena PK (1997). Melatonin in feverfew and other medicinal plants. Lancet.

[CR44] Murch SJ, Krishnara JS, Saxena PK (2000) Tryptophan is a precursor for melatonin and serotonin biosynthesis in in vitro regenerated St. John’s wort (*Hypericum perforatum* L. cv. Anthos) Plant Cell Rep 19:698–704 10.1007/s002990000206 30754808

[CR45] Pasqua G, Avato P, Monacelli B, Santamaria AR, Argentieri MP (2003). Metabolites in cell suspension cultures, calli, and in vitro regenerated organs of *Hypericum perforatum* cv Topas. Plant Sci.

[CR46] Pittendrigh CS, Follett DE (1981). Circadian organization and the photoperiodic phenomena. Follett BK.

[CR47] Pittendrigh CS (1993). Temporal organization: reflection of a Darwinian clock-watcher. Annual Rev Physiol.

[CR48] Poeggeler B, Balzer I, Hardeland R, Lerchl A (1991). Pineal hormone melatonin oscillates also in the dinoflagellate *Gonyaulax polyedra*. Naturwissenschaften.

[CR49] Reiter RJ (1996). Functional aspects of the pineal hormone melatonin in combating cell and tissue damage induced by free radicals. Eur J Endocrinol.

[CR50] Reppert SM (1997). Melatonin receptors: molecular biology of a new family of G-protein-coupled receptors. J Biol Rhythms.

[CR51] Robson NKB (2003) *Hypericum* botany. In: Ernst E (ed) Hypericum: The genus *Hypericum*., Taylor & Francis Inc., London and New York

[CR52] Roenneberg T, Deng TS (1997). Photobiology of the *Gonyaulax* circadian system. I. Different phase response curves for red and blue light. Planta.

[CR53] Roenneberg T, Morse D (1993). Two circadian oscillators in one cell. Nature.

[CR54] Roenneberg T, Colfax GN, Hastings JW (1989). A circadian rhythm of population behavior in *Gonyaulax polyedra*. J Biol Rhythms.

[CR55] Roenneberg T, Deng TS, Eisensamer B, Mittag M, Neher I, Rehman J (1995). Cellular mechanisms of circadian clocks. Wien med Wschr.

[CR56] Sack RL, Lewy AJ (1997). Melatonin as a chronobiotic: Treatment of circadian desynchrony in night workers and the blind. J Biol Rhythms.

[CR57] Sack RL, Brandes RW, Kendall AR, Lewy AJ (2000). Entrainment of free-running circadian rhythms by melatonin in blind people. N Engl J Med.

[CR58] Saunders DS (2016). The temporal ‘structure’ and function of the insect photoperiodic clock: a tribute to Colin S. Pittendrigh. Physiol Entomol.

[CR59] Schaefer M, Hardeland R (2009). The melatonin metabolite N-acetyl-5-methoxykynuramine is a potent singlet oxygen scavenger. J Pineal Res.

[CR60] Schempp CM, Krikin V, Simon-Haarhaus G, Kersten A, Kiss J, Termeer CC, Gilb B, Kaufmann T, Borner C, Sleeman JP, Simon JC (2002). Inhibition of tumor cell growth by hyperforin, a novel anticancer drug from St. John’s wort that acts by induction of apoptosis. Oncogene.

[CR61] Tan D-X, Reiter RJ, Manchester LC, Yan MT, El-Sawi M, Sainz RM, Mayo JC, Kohen R, Allegra M, Hardeland R (2002). Chemical and physical properties and potential mechanisms: melatonin as a broad spectrum antioxidant and free radical scavenger. Curr Topics Med Chem.

[CR62] Tan D-X, Hardeland R, Manchester LC, Korkmaz A, Ma S, Rosales-Corral S, Reiter RJ (2011). Functional roles of melatonin in plants, and perspectives in nutritional and agricultural science. J Exp Bot.

[CR63] Tong Q, McGonnell IM, Demmers TGM, Roulston N, Bergoug H, Romanini CE, Verhelst R, Guinebretière M, Eterradossi N, Berckmans D, Exadaktylos V (2018). Effect of a photoperiodic green light programme during incubation on embryo development and hatch process. Animal.

[CR64] Tosini G, Menaker M (1996). Circadian rhythms in cultured mammalian retina. Science.

[CR65] Wever R (1979). The circadian system of man.

[CR66] Zhang J, Wang L, Xie W, Hu S, Zhou H, Zhu P, Zhu H (2020). Melatonin attenuates ER stress and mitochondrial damage in septic cardiomyopathy: a new mechanism involving BAP31 upregulation and MAPK-ERK pathway. J Cell Physiol.

[CR67] Zhao D, Yu Y, Shen Y, Liu Q, Zhao Z, Sharma R, Reiter RJ (2019). Melatonin synthesis and function: evolutionary history in animals and plants. Front Endocrinol.

[CR68] Zisapel N (2018). New perspectives on the role of melatonin in human sleep, circadian rhythms and their regulation. Br J Pharmacol.

