# Supplementary figures and images for: Effects of circadian clock and light on melatonin concentration in Hypericum perforatum L. (St. John’s Wort)
Source: Bot Stud. 2020 Sep 15;61:23. doi: 10.1186/s40529-020-00301-6 (PMC7492311; doi:10.1186/s40529-020-00301-6)

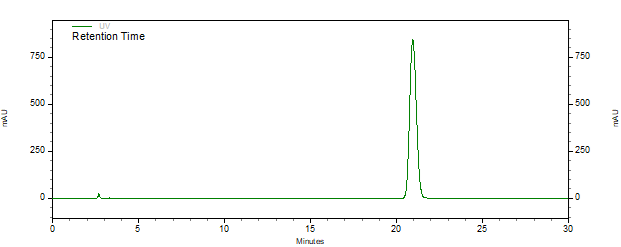

Supplement: Supplementary file 1 — Additional file 1: Figure S1. HPLC analysis of melatonin standard. [file 40529_2020_301_MOESM1_ESM.png]

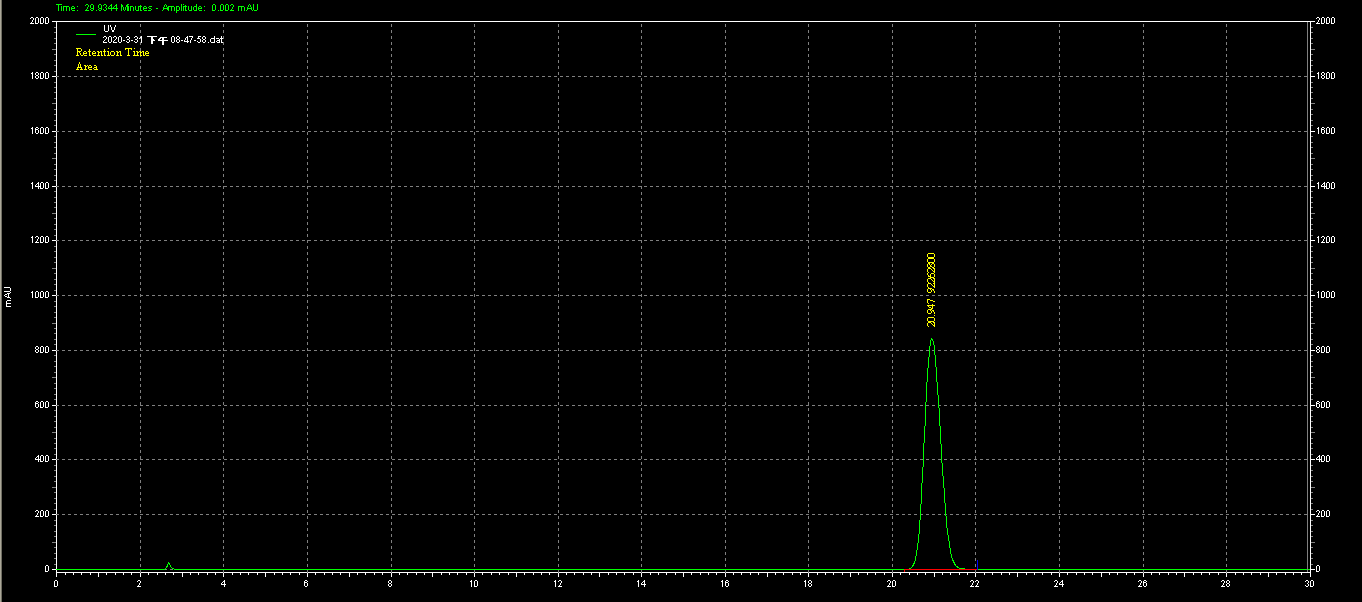

Supplement: Supplementary file 2 — Additional file 2: Figure S2. UV spectrum for melatonin standard in HPLC. [file 40529_2020_301_MOESM2_ESM.png]
